# Antibody-Positive Type 1 Diabetes in a Family With a Pathogenic *HNF1A*-MODY Variant and Variable Age of Onset

**DOI:** 10.1210/jcemcr/luaf229

**Published:** 2025-10-07

**Authors:** Michael E McCullough, Anisa M Dye, Balamurugan Kandasamy, Siri Atma W Greeley, Rochelle N Naylor, Louis H Philipson

**Affiliations:** Section of Adult and Pediatric Endocrinology, Diabetes and Metabolism and Kovler Diabetes Center, University of Chicago, Chicago, IL 60637, USA; Section of Adult and Pediatric Endocrinology, Diabetes and Metabolism and Kovler Diabetes Center, University of Chicago, Chicago, IL 60637, USA; Section of Adult and Pediatric Endocrinology, Diabetes and Metabolism and Kovler Diabetes Center, University of Chicago, Chicago, IL 60637, USA; Section of Adult and Pediatric Endocrinology, Diabetes and Metabolism and Kovler Diabetes Center, University of Chicago, Chicago, IL 60637, USA; Section of Adult and Pediatric Endocrinology, Diabetes and Metabolism and Kovler Diabetes Center, University of Chicago, Chicago, IL 60637, USA; Section of Adult and Pediatric Endocrinology, Diabetes and Metabolism and Kovler Diabetes Center, University of Chicago, Chicago, IL 60637, USA

**Keywords:** monogenic diabetes, MODY, *HNF1A*, *HNF1A*-MODY, diabetes, type 1 diabetes

## Abstract

Monogenic diabetes (MD) is a relatively rare and heterogeneous group of disorders caused by pathogenic single-gene variants or abnormalities resulting in hyperglycemia. MD represents approximately 3.5% of all diabetes cases diagnosed before age 35 years, though it is possible for MD to develop at later ages. MD diagnoses have implications for precision therapy and cascade genetic testing. A hallmark characteristic suggesting MD is a multigenerational family history of nonobese diabetes diagnosed before age 35 with an autosomal dominant inheritance. However, even with a known family history of genetically confirmed MD, it is possible for an individual within that family to have a different form of diabetes. Here, we present a case from the University of Chicago Monogenic Diabetes Registry of an individual with antibody-positive type 1 diabetes in a family with a history of a genetically confirmed known pathogenic *HNF1A* variant causing maturity-onset diabetes of the young (MODY) with variable age of onset in affected individuals. This family pedigree showcases that *HNF1A*-MODY can develop at any age and illustrates the importance of every individual receiving a thorough work-up for accurate diabetes classification, including obtaining antibody testing and genetic testing, when indicated, to provide optimal treatment and management.

## Introduction

Monogenic diabetes (MD) is a relatively rare and heterogeneous group of disorders caused by an increasing number of pathogenic single-gene variants or abnormalities that cause disruptions in glycemia. It is estimated that collectively MD may represent up to 3.5% of all cases of diabetes diagnosed before age 35 years [1-6], though it is possible for onset to occur at even older ages. MD is distinct from the more common forms of diabetes, type 1 (T1D) and type 2. Each form of diabetes has a unique etiology and pathophysiology, and treatment recommendations often differ. Specifically for MD, there are implications for precision therapy, other co-occurring medical conditions, and risk of recurrence to offspring. There are several clinical features that can suggest MD. Among others, these can include diagnosis before age 35, nonobese, negative diabetes autoantibodies, detectable c-peptide, lack of signs of insulin resistance, and autosomal dominant inheritance pattern. When a child presents with hyperglycemia and has a parent with genetically confirmed MD, it may be assumed that the child inherited the genetic variant from their parent. However, a thorough workup for diabetes classification and genetic testing in the child is still recommended as it is still possible to have an alternative form of diabetes or to have co-occurring diabetes types. Here, we present a case from the University of Chicago Monogenic Diabetes Registry (UCMDR) of a child with new-onset T1D from a family with multiple individuals affected by *HNF1A*–maturity-onset diabetes of the young (MODY) presenting with variable age of onset. This demonstrates it is possible to develop T1D even in the context of a family with known *HNF1A*-MODY and shows that *HNF1A*-MODY can present later in life. This case reinforces the need to complete a thorough work-up for accurate diabetes classification, including checking for diabetes autoantibodies and considering T1D genetic risk scores (T1DGRS) when available, in new-onset diabetes in the setting of families with MD.

## Case Presentation

**Figure 1.**
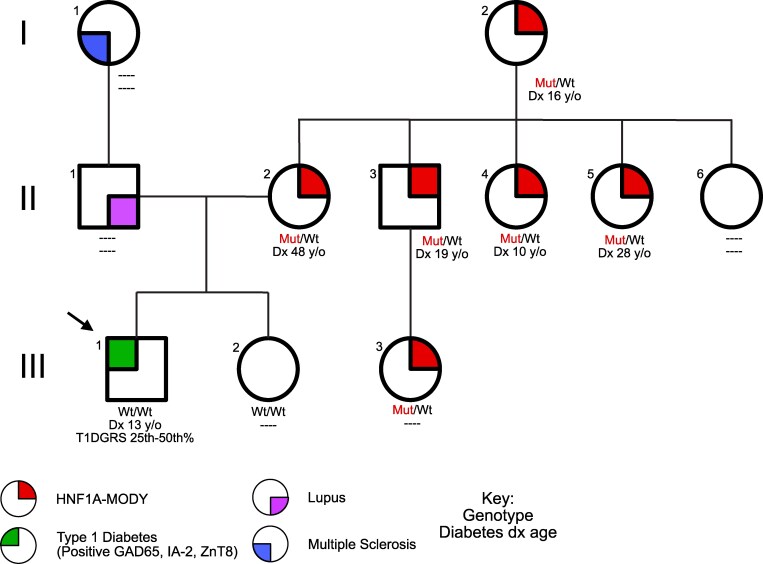
Pedigree of the family. The proband is indicated with an arrow. Affected individuals are shown with a filled symbol. Unaffected participants are shown with unfilled symbols. The genotype and age of diabetes diagnosis are shown below each individual. The type 1 diabetes genetic risk scores (T1DGRS) is also listed for the proband.

A 13-year-old boy with no significant past medical history presented with fatigue, 20-pound (∼9.1-kg) weight loss, polyuria, polydipsia, and hyperglycemia noted on home glucose monitor. His family history was significant for multigenerational genetically confirmed *HNF1A*-MODY due to a heterozygous, pathogenic mutation in the *HNF1A* gene (NM_000545.8 [*HNF1A*]: c.370C > T, p.Gln124*, PVS1, PM2_Supporting, PS4_Moderate). This premature stop codon in the DNA-binding domain has previously been described in a MODY-like proband from a Danish patient [7]. Our patient’s mother, maternal uncle, first maternal cousin, 2 maternal aunts, and maternal grandmother all carry the same aforementioned *HNF1A* variant. Of note, his mother was diagnosed with diabetes at age 48, which is far beyond the mean diagnosis age for *HNF1A*-MODY of 20.3 years [8]. His father has lupus, and his paternal grandmother has multiple sclerosis (pedigree shown in Fig. 1). He was not on any medications at the time of presentation. At the time of diabetes diagnosis, his height was 5 feet 2 inches (∼157.5 cm), and his weight was 88 pounds (∼40 kg) (body mass index [BMI] 16.1, 11th percentile for age; prior to weight loss, BMI was approximately 80th percentile). Despite the multigeneration history of MD, the clinical presentation and paternal family history of autoimmunity prompted a thorough investigation for diabetes classification.

## Diagnostic Assessment

The laboratory parameters at presentation included blood glucose 480 mg/dL (SI: 26.7 mmol/L) (reference range < 200 mg/dL [SI: < 11.1 mmol/L]) and glycated hemoglobin A_1c_ (HbA_1c_) 14% (SI: 130 mmol/mol) (reference range < 6.5% [SI: < 48 mmol/mol]). There was no diabetic ketoacidosis. Diabetes autoantibody testing revealed positive islet antigen-2 (>120 unit/mL, reference, 0-7.4 unit/mL), positive glutamic acid decarboxylase-65 (70.3 unit/mL, reference 0-5.0 unit/mL), and positive zinc transporter 8 autoantibodies (>500 unit/mL, reference 0-15.0 unit/mL). Research-based genetic testing and a T1DGRS were performed by the UCMDR. Single-gene Sanger sequencing of *HNF1A* in the proband was negative for the known HNF1A family variant. T1DGRS revealed a risk in the 25th to 50th percentile. A diagnosis of type 1A diabetes was made for this child, and insulin therapy was started.

## Treatment

At time of last follow-up (7 months after diagnosis), he was maintained on a multiple daily injection regimen using long-acting and rapid-acting insulin. His average total daily insulin dose was 32 total units (0.71 u/kg/d), and he was using a continuous glucose monitor.

## Outcome and Follow-up

Diabetes was well-controlled with follow-up HbA_1c_ performed 3 months after diagnosis of 6% (SI: 42 mmol/mol).

## Discussion

This case demonstrates the possibility for occurrence of T1D independently in a family with a strong history of genetically confirmed pathogenic *HNF1A*-MODY. A similar case of T1D and *HNF1A-*MODY coexisting in the same family has been reported previously in the United Kingdom [9]. This underscores the critical importance of obtaining a correct diabetes diagnosis, which guides therapy, informs recurrence risk in offspring, and can direct cascade genetic testing and screening for other medical conditions. As such, it is strongly recommended to pursue a thorough work-up even if there is a family history of MODY as this case demonstrates it cannot be assumed that newly diagnosed diabetes is MODY. Clinical features such as diabetes autoantibody status can assist with a correct diagnosis. Nevertheless, if the family history warrants, genetic testing should still be considered even in the presence of positive diabetes autoantibodies, given the possibility for co-occurring T1D and MODY in the same individual. Lastly, it is notable that the age of diabetes diagnosis among family members with *HNF1A*-MODY ranged from 10 to 48 years. This demonstrates that despite the high penetrance of pathogenic *HNF1A* variants in family cohorts (86% of family members have diabetes diagnosed by age 40 years [10]), the age of onset can be highly variable.

## Learning Points

- A strong family history of monogenic diabetes should not preclude a thorough work-up for alternative or co-occurring forms of diabetes.
- Accurate classification of diabetes has important implications for therapy, long-term prognosis, and genetic counseling, including recurrence risk and family screening.
- The age of diagnosis of *HNF1A*-MODY can be highly variable, even within the same family, and sometimes occurs many years beyond the usual cutoff of 35 years.
- There is potential utility in using T1DGRS to give insight into the underlying diagnosis, especially if there are features or history suggestive of multiple possible etiologies. However, there is no score that is definitive for T1DM diagnosis.

## Data Availability

Restrictions apply to the availability of some or all data generated or analyzed during this study to preserve patient confidentiality or because they were used under license. The corresponding author will on request detail the restrictions and any conditions under which access to some data may be provided.

## Abbreviations

BMI: body mass index
HbA_1c_: glycated hemoglobin A_1c_
MD: monogenic diabetes
MODY: maturity-onset diabetes of the young
T1D: type 1 diabetes
T1DGRS: type 1 diabetes genetic risk scores
UCMDR: University of Chicago Monogenic Diabetes Registry
